# Automated Matchmaking of Researcher Biosketches and Funder Requests for Proposals Using Deep Neural Networks

**DOI:** 10.1109/access.2024.3427631

**Published:** 2024-07-15

**Authors:** SIFEI HAN, RUSSELL RICHIE, LINGYUN SHI, FUCHIANG TSUI

**Affiliations:** 1 Tsui Laboratory, Department of Biomedical and Health Informatics, Children’s Hospital of Philadelphia, Philadelphia, PA 19146, USA; 2 Cognitive Science and MindCORE, University of Pennsylvania, Philadelphia, PA 19104, USA; 3 Department of Anesthesiology and Critical Care, Perelman School of Medicine, University of Pennsylvania, Philadelphia, PA 19104, USA; 4 Department of Biostatistics, Epidemiology, and Informatics, Perelman School of Medicine, University of Pennsylvania, Philadelphia, PA 19104, USA

**Keywords:** Document similarity, deep neural networks, data augmentation

## Abstract

This study developed an automated matchmaking system using deep neural networks to enhance the efficiency of pairing researcher biosketches with funders’ requests for proposals (RFPs). In thus U.S., with over 900 federal grant programs and 86,000+ foundations, researchers often spend up to 200 hours on each application due to low success rates, forcing them to apply multiple times a year. Our approach improves on existing systems by fixing issues like unreliable keyword searches, and one-size-fits-all recommendations. We analyzed 12,991 biosketches from a research institution and 2,234 RFPs from the National Institutes of Health, spanning 2014 to 2019. Employing four advanced deep-learning models, utilizing cross and Siamese encoding strategies, we benchmarked their performance against conventional predictive models such as logistic regression and support vector machines. The most effective model integrated BERT with cross-encoding, a post-BERT BiLSTM layer, and back translation (BC2BT), achieving an F1-score of 71.15%. These results demonstrate the potential of sophisticated natural language processing techniques to automate complex matchmaking tasks in the research funding sector. This approach not only improves the precision of matching researchers to suitable funding opportunities but also sets a promising foundation for future advancements in automated funding mechanisms.

## 1. INTRODUCTION

Writing grant applications is time-consuming, often taking between 80 and 200 hours per application [1]. Low grant award rates [2] compound this issue, forcing researchers to apply for multiple grants annually to secure adequate funding. This situation highlights the need to improve efficiency in the grant application process. We focus on improving matchmaking between researchers and requests for proposals (RFP’s).

Currently, the research grant application landscape is vast, with over 900 federal grant programs offered by 26 different grant-making agencies, and 86,203 grant-awarding foundations in the United States in 2015 [1]. This complexity makes it challenging for researchers to identify grant programs best aligned with their work and most likely to fund them. Automated matchmaking systems exist but have three limitations. First, (L1) current systems for matching researchers with RFPs (often implemented in commercial solutions like Pivot [3] or Elsevier Pure [4]) utilize variations on keyword search, which is notoriously brittle (e.g., a researcher keyword of ‘chemo’ will not match an RFP with ‘cancer’). Second, (L2) automatic systems are often used at the departmental level, providing the same recommended grant list to all the researchers in a department, even if they have varying research interests, skills, resources, etc. Third, to our knowledge, and most importantly, (L3) there are no published works evaluating such RFP-researcher matching systems.

This work makes the following contributions by addressing the aforementioned shortcomings. First, we use deep neural networks to perform RFP-researcher matching (addressing L1). Deep neural networks have achieved state of the art (well beyond keyword-based approaches) in many related natural language processing tasks, like document similarity [5]. Because deep learning systems treat words as vectors such that similar words (‘cancer’ and ‘chemo’) will have similar vectors, a deep learning system can (unlike simple keyword matching) recognize the match between an RFP with ‘cancer’ and a biosketch with ‘chemo’. Second, our approach uses researcher biosketches (or biographical sketches), commonly used in grant submissions, to represent interests at the user-level (addressing L2). Third, we developed an innovative model that integrates a Bidirectional Encoder Representations from Transformers (**B**ERT) model [6] with a **C**ross-encoding strategy, supplemented by a **B**i-LSTM layer, and employed **B**ack **T**ranslation (BC2BT) for data augmentation purposes; we compared this approach with other approaches utilizing BERT and post-BERT layers, as well as with traditional machine learning approaches based only on keywords. Last, we are the first to develop a deep-learning-based RFP-research matching system, which moves beyond traditional keyword-based search methods. (addressing L3). We hypothesize that using deep learning can provide accurate matchmaking between researchers’ biosketches and funding opportunities. Our proposed personalized grant recommendation system [7] could potentially save researchers and funding agencies thousands of hours in matching with each other, and may eventually be generalized to related problems of matching workers to opportunities (e.g., grant or manuscript reviews, job positions, etc.)

## II. REVIEW OF RELATED WORKS

With a lack of published studies on RFP-researcher matching, the most closely related work is matching job postings with resumes. The study [8] investigates various machine learning classifiers for an automated resume recommendation system, including Random Forest, Multinomial Naive Bayes, Logistic Regression, and Linear Support Vector Classifier, with accuracy ranging between 38.99% and 78.53%. The paper [9] presents a novel job-resume matching network that employs a multi-view co-teaching network approach to tackle the challenges of sparse interaction data, utilizing text and relationship-based models to refine the matching process. This approach attained F1-score between 66.1% and 66.9% based on the job type. Furthermore, the review [10] highlights 18 related studies on resume-job matching. The techniques they used include Collaborative Filtering, TFIDF vectorization, Random Forest, Naïve Bayes, Logistic Regression, SVM, K-NN, BiLSTM, XG Boost, and Cosine/Jaccard Similarity. Therefore, we decided to use commonly used Logistic Regression and SVM as baseline methods in our study.

## III. MATERIAL AND METHODS

This section describes the corpus of this study. The Institutional Review Board at the Children’s Hospital of Philadelphia (CHOP) reviewed the study and determined this to be exempt research (IRB 23–021339).

### A. DATASET: RFP-BIOSKETCH PAIRS

**FIGURE 1.**
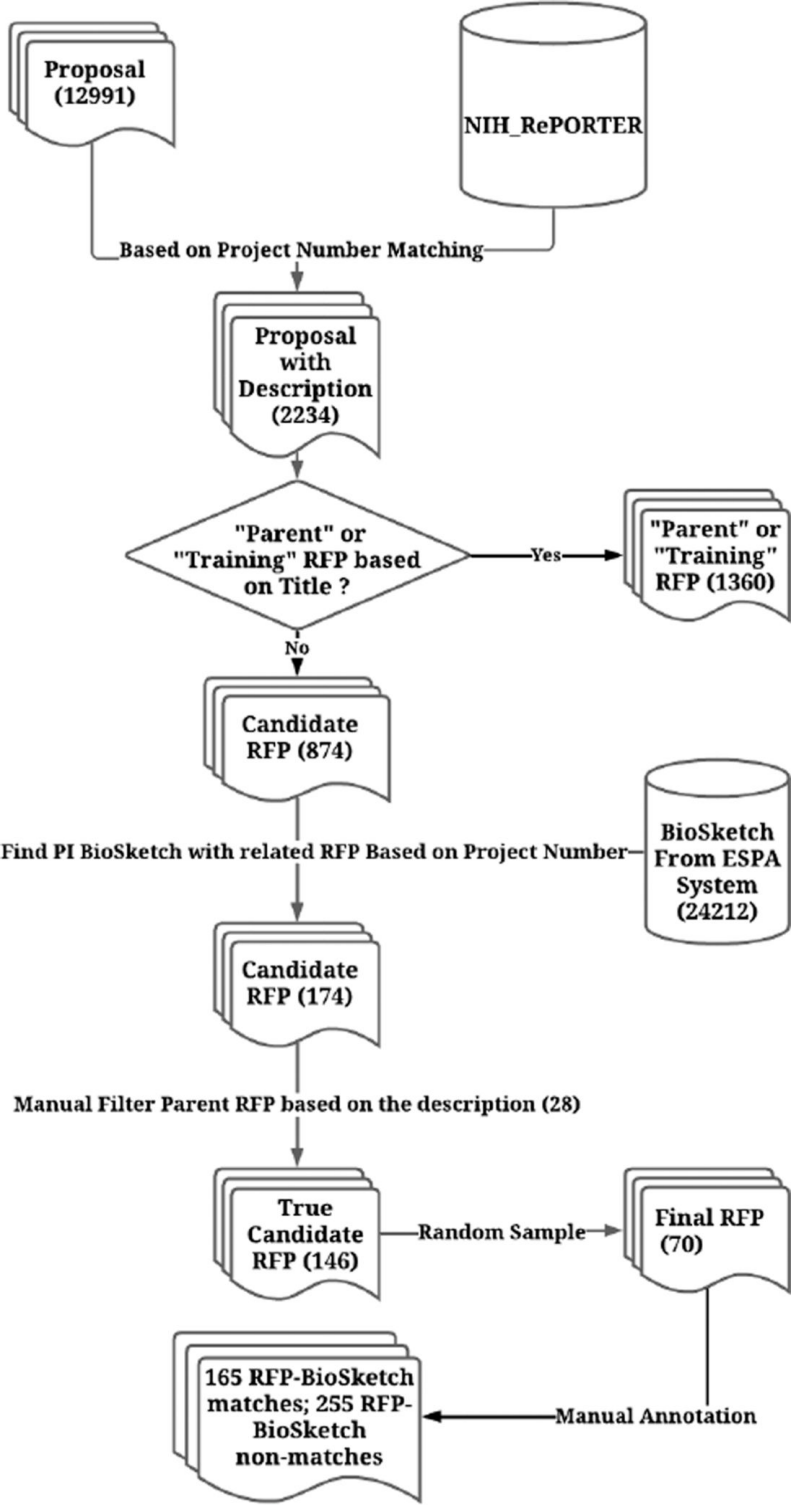
Dataset curation flowchart.

Figure 1 summarizes our dataset curation process. We first retrieved all the submitted grant proposals (n=12,991) from CHOP between 2004 and 2019. We then selected just proposals submitted to the National Institute of Health (NIH). For those proposals with a funded project number (a unique identifier for a request for proposal from NIH), we retrieved the text of the RFP from NIH RePORTER [11]. This yielded 2,234 RFPs. Next, we removed two types of RFPs from the dataset based on RFP titles: 1) parent RFPs, which are broad funding opportunities permitting research on nearly any topic, and 2) training grants, which are awards to support training of pre- and post-doctoral researchers. We removed these two RFP types since parent RFPs are too broad to match any researchers in particular, and training grants are not necessarily research-focused. This resulted in 1,360 RFPs being removed, with 874 RFPs remaining. Among these 874 RFPs, we used CHOP’s biosketch [12] database to find the PI biosketches that were used to apply to any of these RFPs (as indicated by matching project numbers), which yielded 174 RFP-biosketch matches. Finally, we manually reviewed these 174 RFPs and removed 28 additional parent RFPs which did not indicate their parent status in their title. The final dataset, therefore, has 146 RFP-biosketch matches.

These 146 RFP-biosketch pairs are cases where the PIs were awarded funding, which we consider as positive (match) or case biosketch samples. To keep manual annotation feasible, we randomly selected a subset of the 146 pairs (n=70 RFPs) for the study corpus. To include negative (non-match) or control biosketch samples in the corpus, we randomly selected 350 RFP-biosketch pairs from the biosketch database at CHOP, i.e. five biosketches for each of these 70 RFPs.

We emphasize that by manually annotating gold standard matches between RFP’s and individual research biosketches, we address both limitations L2 (that automatic matchmaking systems are often used at the departmental not individual level) and L3 (that previous matchmaking systems have not been evaluated against a ground truth).

### B. RFP-BIOSKETCH PAIR ANNOTATION

Since the control biosketches were selected randomly, some of them could be qualified for the RFPs. To detect this, two librarians initially annotated control biosketches’ suitability for their assigned RFPs, scoring them as either a non-match (0) or match (1). Initially, we annotated five RFPs with five control biosketches each, resulting in 25 annotated RFP-biosketch pairs. This phase showed an inter-annotator agreement (Cohen’s Kappa [13]) was 88.37%. Disagreements were handled by discussion. As the Kappa-score indicated that the annotators had an almost perfect agreement, we asked the two annotators to individually annotate 5 control biosketches for each of 30 RFPs, creating 150 pairs. We then used an additional 5 RFPs (each with 5 controls, for 25 RFP-biosketch pairs) to re-measure annotation quality. By combining these with the original 5 RFPs, we had 50 RFP-biosketch pairs annotated by both librarians; we obtained an overall Kappa-score of 52.63% (moderate agreement).

Further assessment was done by a third annotator (co-author RR) who reviewed all 350 RFP-biosketch pairs, with agreement levels with two librarians was 46.23% and 54.55%, respectively. We consider reasons for, and implications of, this moderate agreement in the discussion. We ultimately annotated five random biosketches for each of the 70 RFPs, leading to 350 annotated RFP-biosketch pairs. Matches were combined with pre-identified positive cases, totaling 165 matches, while the rest were considered as 255 non-matches (control samples), leading to 415 labeled RFP-biosketch pairs. We focused mainly on the “Personal Statement” of biosketches and the “Funding Opportunity Description” of RFPs. The implications of this selective focus are considered in the discussion section.

### C. DEEP LEARNING ARCHITECTURE AND MODELS

**FIGURE 2.**
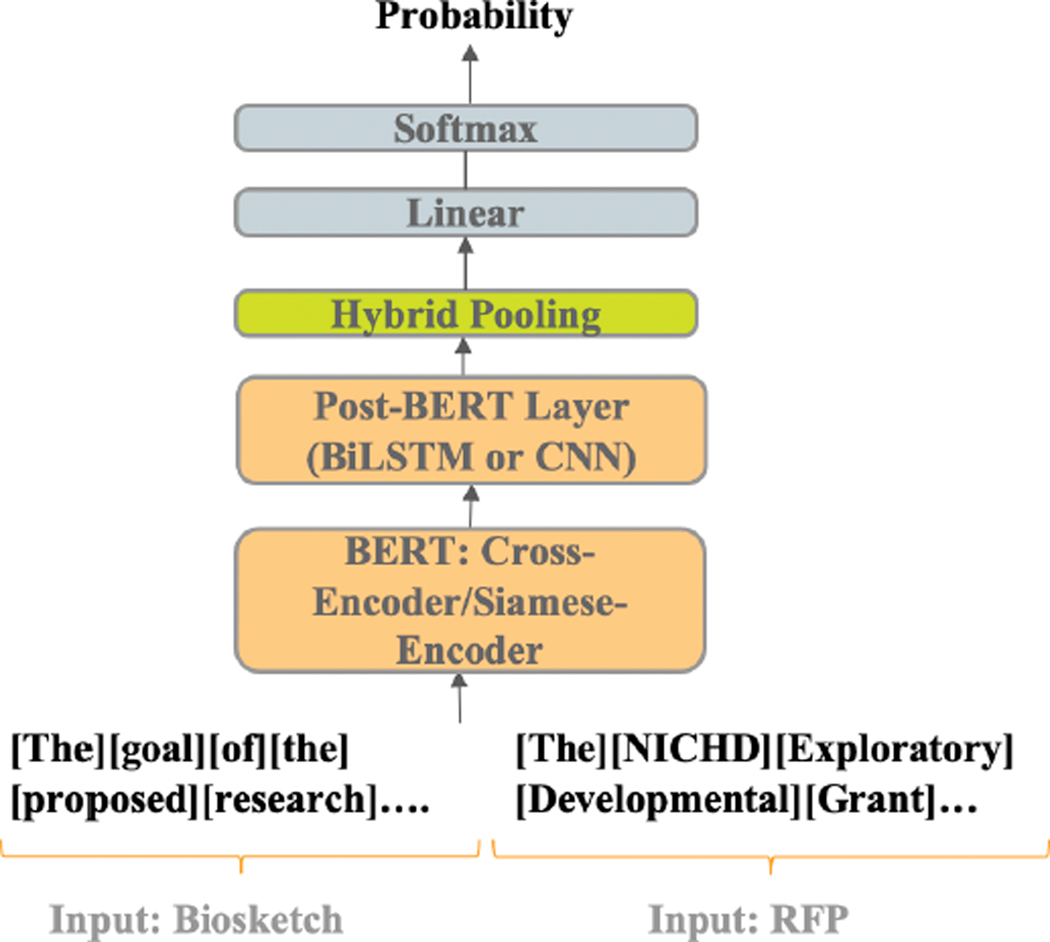
The overall system architecture for measuring similarity between two documents: A researcher’s biosketch and a request for proposal (RFP). Hybrid pooling: The pooling layer using both max pooling and average pooling. Linear layer: A fully connected layer serves to map the learned representations from the previous layers to a space where they can be effectively separated or classified. Softmax layer: A type of activation function that converts the output of the linear layer into a probability distribution.

We developed four deep neural network models (Table 1). For each model, we fine-tuned a pre-trained BERT (base with 110M parameters), a transformer that has achieved state-of-the-art in many NLP tasks, including document-pair classification tasks closely related to our RFP-biosketch matching task, e.g., duplicate detection and textual entailment [14]. Previous work has shown that combining transformers with other DNN architectures improves performance in tasks like machine translation [15]. A previous study [16], [17] shows the success that different encoder approach with additional layers on short text. Therefore, we tested both cross-encoding [18] and Siamese encoding [17] strategies, with post-BERT CNN or Bi-LSTM layers followed by a hybrid pooling layer (max pooling plus average pooling) on longer documents. Our four models thus follow a two (Siamese encoding vs cross-encoding) by two (Bi-LSTM layer on top of BERT vs CNN layer on top of BERT) structure (Table 1). Figure 2 illustrates the framework common to all four models.

### D. BERT CROSS-ENCODER

**FIGURE 3.**
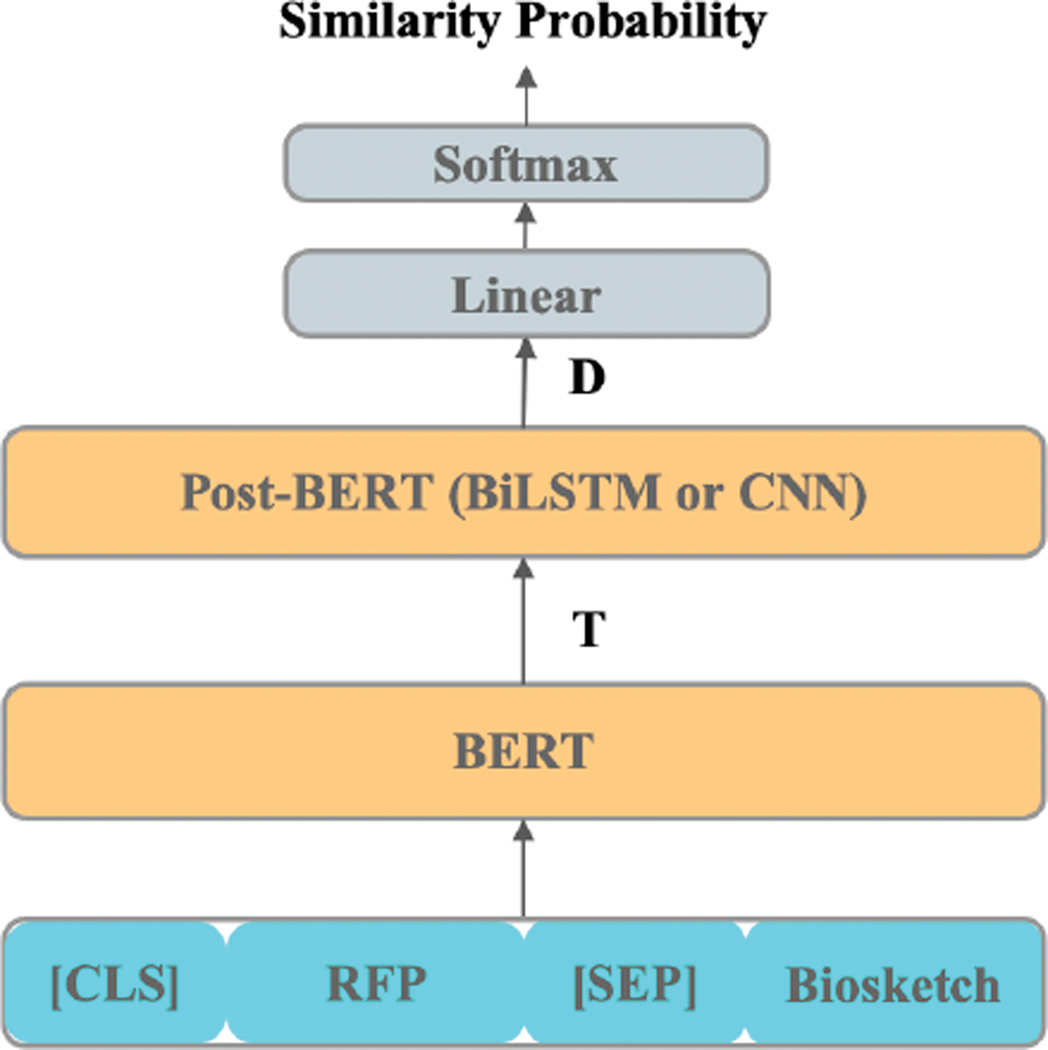
Cross-encoding with BERT and a Post-BERT (BiLSTM or CNN) layer. T is a (N+1,d)-shaped matrix containing d-dimensional contextualized BERT embeddings for each of the N tokens in the combined RFP and biosketch and the special [SEP] token separating the RFP and biosketch. D is a vector summarizing the RFP-biosketch pair. The special token [CLS] is conventionally pre-pended to the entire input documents. The vector for this token in higher layers of a network is typically used to represent an entire sequence for classification (hence CLS).

In cross-encoding [6] (Figure 3), a single BERT network is provided the sequence [RFP + [SEP] + Biosketch], and produces contextualized representations for each token in the RFP and biosketch, as well as for the special [SEP] tokens. The [SEP] token encodes the boundary between the RFP and biosketch. This produces the sequence of contextualized embeddings, $T=\left[t_{0},t_{1},\cdots ,t_{N}\right]$ where N is the number of words in the RFP and biosketch (T therefore contains N+1 tokens, which includes the [SEP] token). This sequence T is fed to a post-BERT layer, either a CNN or LSTM. The token-level outputs of this layer are average pooled and max pooled, and then concatenated, to produce a summary representation, D, of the combination of the RFP and the biosketch. A softmax classifier then uses D to predict whether the RFP-biosketch pair is a match or not.

### E. BERT SIAMESE-ENCODER

**FIGURE 4.**
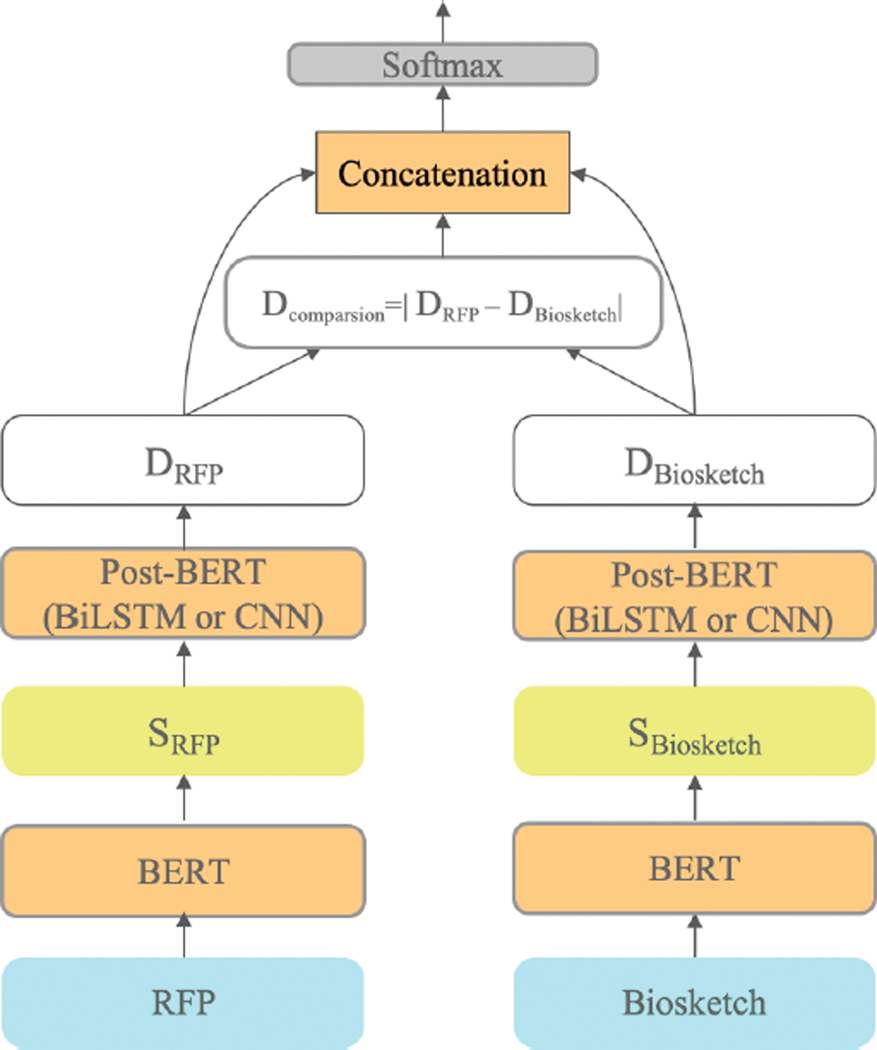
Siamese-encoding with BERT and a Post-BERT (BiLSTM or CNN) layer. $S_{RFP}$ and $S_{biosketch}$ are (N,d)- and (M,d)-shaped matrices (respectively) containing d-dimensional contextualized BERT embeddings for each of the N or M tokens in the RFP or biosketch, respectively. $D_{RFP}$ and $D_{Biosketch}$ are vectors summarizing the RFP and biosketch.

The Siamese encoding network [19] (Figure 4), uses two identical BERT networks to encode two documents (biosketch and RFP) separately, whereas the cross-encoding network uses a single BERT network to encode one document comprising the two concatenated documents. The representations from each network are then compared to classify the relationship between the documents. In the present case, this means that the text of the RFP and the biosketch are each passed separately to BERT, producing sequences of contextualized token representations $S_{RFP}$ and $S_{biosketch}$. $S_{RFP}$ and $S_{biosketch}$ are then passed separately to identical post-BERT layers (CNN or LSTM) which also share weights. Average pooling and max pooling are applied to these post-BERT layers’ outputs, and these poolings are concatenated to obtain separate summary representations for the biosketch, $D_{biosketch}$ and the RFP, $D_{RFP}$. We then compute the elementwise absolute value of the difference between $D_{biosketch}$ and $D_{RFP}$ to obtain the vector $D_{comparison}$. Finally, a softmax classifier then uses the concatenation of $D_{RFP}$, $D_{Biosketch}$, and $D_{comparison}$ to predict whether the RFP-biosketch pair is a match.

### F. POST-BERT LAYERS

BERT produces contextualized token-level vector representations. More specifically, the cross-encoding strategy produces T for the combination of the RFP and biosketch (Figure 3), while the Siamese encoding strategy produces $S_{RFP}$ and $S_{biosketch}$ for the RFP and biosketch (Figure 4), respectively. These representations are then passed to either a BiLSTM or CNN for further processing. To generalize the following descriptions of BiLSTM and CNN over the two BERT encoding strategies, we considered a token-level BERT representation, R, which refers to T in the case of cross-encoding, and $S_{RFP}$ or $S_{biosketch}$ in the Siamese encoding, where $r_{0}$ is the embedding for the first token in R; $r_{\text{t}}$ is the embedding for the t-th token in R.

### G. BILSTM

**FIGURE 5.**
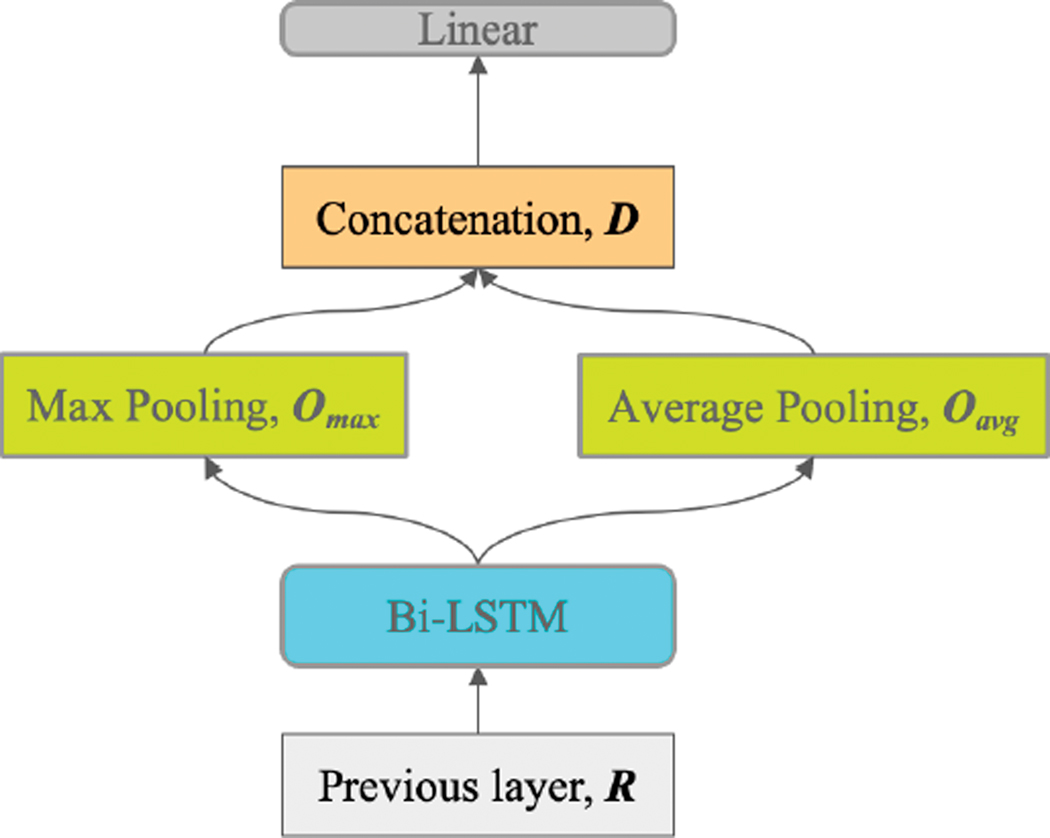
Post-BERT layer: Bi-LSTM feature extraction.

We employed a bidirectional LSTM [20], which used one LSTM to process the input from the first token to the last and another LSTM to process the input from the last token to the first. Typically, in a bidirectional LSTM, $h_{t}$ of the last token of the forward LSTM and $h_{t}$ of the first token of the backwards LSTM are concatenated to produce a representation $o_{t}$. Next, we average pool and max pool $o_{t}$, yielding $o_{avg}$ and $o_{max}$, and concatenate these to obtain $D=\left[o_{avg},o_{max}\right]$. D is then used as appropriate for the cross-encoding or Siamese encoding strategies. Next, we use a linear layer to extract features from D which can empower our network’s ability to classify the extracted features (Figure 5).

### H. CNN

**FIGURE 6.**
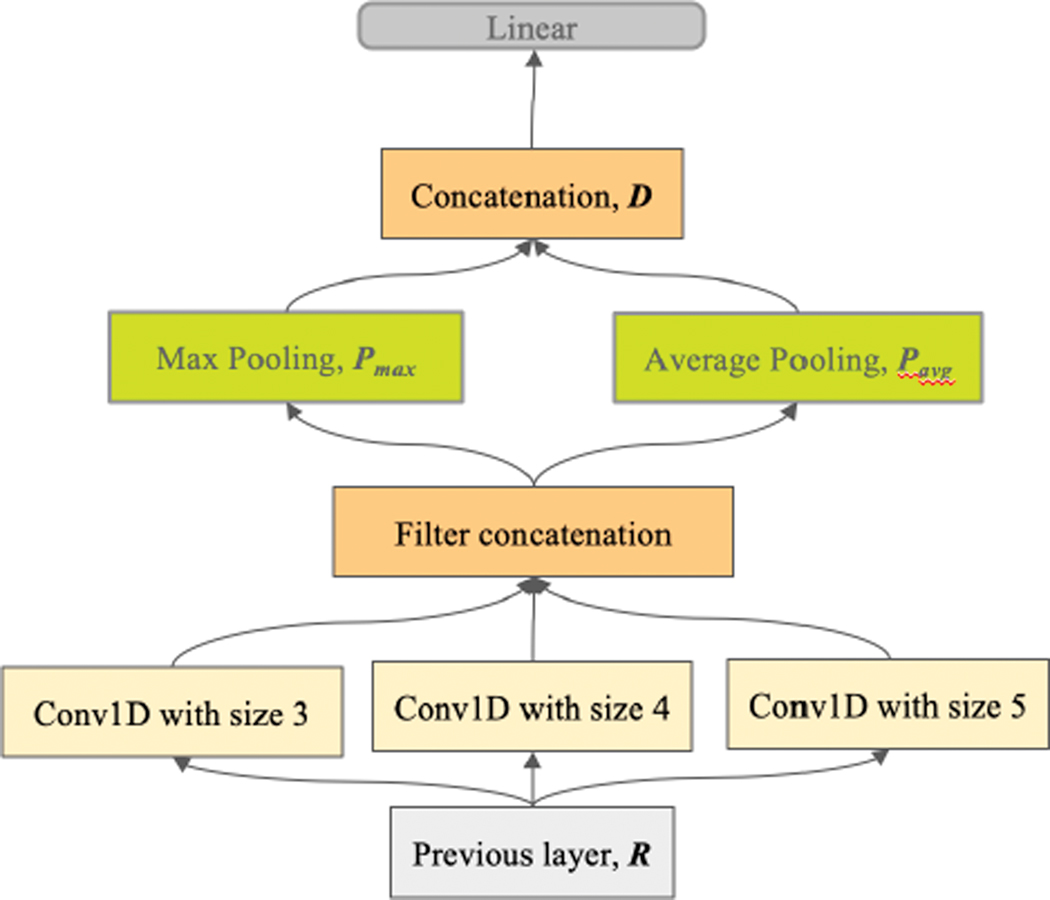
Post-BERT layer: CNN feature extraction.

CNNs [21] employ a convolution operation over the BERT output matrix, R, to produce a feature map. The goal is to learn multiple *convolution filters* (CFs) that can collectively capture diverse representations of the same document. Choosing k filters results in k corresponding feature maps $v^{1},\cdots ,v^{k}$. Different sets of k CFs are learned for different window sizes. The window sizes are parameterized as a sequence $h_{1},\cdots ,h_{H}$ of H unique sizes. Suppose $p^{h_{i}}$ denotes the feature vector produced on k filters with a window size of $h_{i}$, then the final kH×1 feature vector is $p^{*}=p^{h_{1}}∥\cdots ∥p^{h_{H}}$ where || is the vector concatenation operation. Next, we average pool and max pool $p^{*}$, yielding $p_{avg}$ and $p_{max}$, and concatenate these to obtain $D=\left[p_{avg},p_{max}\right]$. D is then used as appropriate for the cross-encoding or Siamese encoding strategies. Next, we feed D into a linear layer to empower our network’s ability to classify the extracted features (Figure 6).

### I. BASELINE MODELS: LOGISTIC REGRESSION (LR) AND SUPPORT VECTOR MACHINE (SVM)

There is no previous study on, or baseline for, matching biosketches with RFP’s. We therefore created our own baseline for comparison to deep neural networks. For text pre-processing, we first removed stopwords from the NLTK [22] stopword list, removed non-ASCII characters, and converted contractions with ‘ into full terms (e.g., replacing “*what’s*” to “*what is*”). Next, we applied TF-IDF feature weighting on the bag-of-words. We also calculated the percentage of (1) word types in common between the RFP and biosketch, (2) common stopword types, and (3) common word tokens; (4) whether the last word of both texts is the same; (5) whether first word of both text is same; and (6) the average token length of both documents. The TF-IDF vector for the RFP+biosketch was concatenated to these 6 features to create a single feature vector for the RFP-biosketch pair. We also tried including the elementwise difference vector between TF-IDF weighed word2vec feature representation, but it proved unhelpful, so we omitted it from our baseline model. For classifiers, we use LR [23] and SVM [24] from Python’s Sci-kit Learn machine learning package [25].

### J. DATA AUGMENTATION

Our dataset has only 420 RFP-Biosketch pairs, which is much smaller than the dataset used in related work on, e.g., matching resumes to job descriptions (n=270K resume-job description pairs). To address this limitation, we employed a widely-adopted strategy known as data augmentation, [26], [27] specifically utilizing the python NLPAug package [28]. This package involves back translation [29], a technique that has demonstrated substantial effectiveness in enhancing longer documents and various NLP tasks [27], [30], [31]. Back translation operates by translating existing samples into a different language, and then translates them back to the original language, such as English, while retaining the original label of the sample. For a given sample x, this process generates a new sample $x^{\prime }$ with lexical and syntactic structure slightly different from x, but keeps the same overall gists and labels. For machine back translation, we used pre-trained English↔German and English↔Russian models from Facebook (wmt19-en-de, wmt19-de-en, wmt19-en-ru, and wmt19-ru-en), which demonstrated accurate translations [15]. For each RFP-Biosketch pair, we performed one back translation to create another 420 RFP-Biosketch pairs. For example, “Exercise benefits the body in many ways.” is translated into German as “*Sport tut dem Körper in vielerlei Hinsicht gut*”, which translates back to English as “*Sport is good for the body in many ways.*”

### K. MODEL CONFIGURATION, TRAINING, AND TESTING

We employed a grid search technique to determine the best batch size, dropout rate, and filter sizes. Both CNN and Bi-LSTM models were trained using the *Adam optimizer* with a common *learning rate* set to *0.00001*, the *decay rate of the moving-average gradient (beta1)* set to *0.9*, and the *decay rate of the squared gradient (beta2)* set to *0.999*. In all model combinations, training was carried out in two stages. In the first stage, which lasted 30 epochs, we froze BERT parameters, and only trained the post-BERT layers. In the second stage, which lasted another 30 epochs, we unfroze BERT parameters; thus, both BERT and post-BERT layers were trained. We used a *mini-batch size of 8*. To avoid overfitting, we used dropout [32], where the *dropout value* was set to *0.3.* For the CNN model [33], we used *filter widths of size 3, 4, and 5*, with *128 filters learned for each size*. In the Bi-LSTM model, we used a *hidden state size of 128*. Hidden units used a *hyperbolic tangent activation function*, and output units used *softmax function*. RFP-biosketch pairs were classified as a “match” when the model’s output probability exceeded the *threshold of 0.5*. We followed a commonly used nested 10-fold cross-validation for model building and testing [34], [35]. We divided the data into 10 partitions stratified by RFP-biosketch pairs. We used 90% of the data (9 partitions) for training models and tested them on the remaining 10% hold-out set. This process was repeated 10 times. To avoid potential bias, the partition was based on RFPs; in other words, the training and test folds did not have overlapped RFPs.

### L. EVALUATION METRICS

The evaluation metrics for models include F1-score, [36] precision (P), and recall (R). F1-score is defined as the harmonic mean between precision (P) and recall (R):

$$P=\frac{TP}{TP+FP},$$

$$R=\frac{TP}{TP+FN},$$

$$F1=\frac{2*P*R}{P+R},$$

where TP, FP and FN are the true positives, false positives, and false negatives, respectively.

### M. SECONDARY STUDIES

We performed secondary studies including ablation and error analysis. The ablation study evaluated each of the three components (BERT layer, feature extraction layer, and hybrid pooling layer) to the impact of the best model’s classification performance. We measured performance impact based on F1 score from 10-fold cross-validation. The feature extraction layer used CNN or BiLSTM. We also tested the large BERT’s (with 340M parameters) impact on the overall performance by replacing the base BERT in the best-performed model. The error analysis focused on identifying and categorizing the types of errors made by the model, such as misclassifications or false positives, to gain insights into areas for further improvement and refinement of the model. This analysis helped in understanding the model’s limitations and provided direction for future enhancements.

## IV. RESULTS

Table 2 summarizes RFP-biosketch matching performance of machine learning models with and without data augmentation. Among the six machine learning models without using data augmentation, CC_BERT_BiLSTM, a cross-encoder BERT, with a BiLSTM layer on top, had best F1 61.46% (95% C.I.: 57.01–65.91%). With data augmentation applied to the best performed CC_BERT_BiLSTM model, the BC2BT model had the best F1 score of 71.15% (63.39–78.91%), which was trained with augmented back translation data using two additional languages, i.e., English↔German and English↔Russian.

For models using different encoding strategies, we found cross-encoding based models (CC_BERT_BiLSTM and CC_BERT_CNN) had improved F1 scores compared to the scores of the Siamese-encoding based models (S_BERT_BiLSTM and S_BERT_CNN) with statistical significance using T-tests (P<0.05). With the same encoding strategy, however, the post-BERT feature extraction approaches using CNN and BiLSTM, e.g., {encoding_strategy}_BERT_CNN and ({encoding_strategy}_BERT_BiLSTM, had similar F1 scores without statistically significant difference (P=0.8480), although BiLSTM based approach had slightly improved performance. Additionally, we further trained and tested the best-performed deep learning model, CC_BERT_BiLSTM, using data augmentation with back translation. The results show that the model augmented with two additional languages (German and Russian) of back translation (BC2BT) outperformed the model without data augmentation (CC_BERT_BiLSTM) in F1 scores (71.15% [66.34–75.96%] vs. 61.46% [58.70–64.22%]) with statistical significance (P<0.01).

### A. ABLATION STUDIES IN THE DEEP NEURAL NETWORKS

Table 3 reports results of an ablation study, which analyzed how each of the three components (BERT layer, feature extraction layer, and hybrid pooling layer) impacts classification performance. We chose the best-performed model BC2BT for the analysis. We trained and evaluated this model under 10-fold cross-validation to analyze the average F1-score loss caused by removing one of the components and using only the BERT large embedding component. We also look at the 95% confidence interval to understand the stability they provide to the training process. First, we found that all components positively contributed to the model performance (F1 score). Without the feature extraction layer (BiLSTM layer), the model’s F1 score dropped by 6.68; without the hybrid pooling that used max and average pooling, the model’s F1 score dropped by 3.43%. When we used the large BERT instead of the base BERT, the F1 score dropped by 1.79%.

### B. RANDOM PAIRS AS NEGATIVE CASES

In contrast to related work on resume-job description matching, we found that treating randomly sampled pairs as controls did not work well (F1=54.5%) It is common in document pair similarity/matching tasks to treat all randomly paired documents as control (non-matched) samples in order to lower the annotation burden [5] We also tried this, which led to 70 RFP-biosketch pairs as matches, and 350 RFP-biosketch pairs as not matches (ratio 1:5). We trained the CC_BERT_BiLSTM model on this dataset. Across 10-fold cross-validation, the average F1-score was 54.52%, with a standard deviation of 9.94%, whereas the same model on our completely annotated dataset reached an F1 of 61.46%, which is about 7% higher. This suggests random pairing to create negative samples works poorly for our task. We speculate on reasons for this in the discussion.

### C. ERROR ANALYSIS

Moreover, we conducted an error analysis on the misclassified RFP-Biosketch pairs. Table 4 shows the confusion matrix generated by BC2BT based on 10-fold cross validation (predictions are generated from each of the 10 test folds).

Among false negatives, we found there are two RFPs that each appeared four times (thus accounting for eight false negatives overall). The first RFP is to identify and promote the development of orphan products for treating rare diseases, which affect fewer than 200,000 people in the United States. Such RFPs could fit many different biomedical domains. Therefore, the annotators believed most physicians/scientists could do such research and apply for the grant. The other RFP’s goal is exploratory/developmental bioengineering research, and it is used to foster the exploration and development of innovative technologies, models, techniques, designs, and methods that have the potential to advance biomedical research.

Among false positives, we found two RFPs, and each appeared three times. The first one covers 15 defined areas to significantly impact biomedical or behavioral science and/or health research. The second one is an exceptionally long document with over 2,800 words on developing the infrastructure and improving the methodology for prospective collection of data from electronic databases containing clinical information.

## V. DISCUSSION

In this study, we built and compared four DL models and two baseline models (logistic regression and SVM) to perform RFP-researcher matching. To our knowledge, we are the first to develop and evaluate a deep-learning-based automated RFP-research matching system. Unlike commercial solutions using keyword-based or non-deep learning approaches, we developed deep learning models to match free-text documents. We found two key findings in this study. First, the two models (CC_BERT_CNN and CC_BERT_BiLSTM) using pre-trained BERT with a cross-encoding strategy outperformed the other models, including two conventional baseline machine learning models and the deep learning models using Siamese encoding strategy (F1 scores: 60.92–61.46% vs. 51.14–56.22%). Second, data augmentation via back translation, e.g., English→{Russian, German}→English, substantially improved model performance by 15.8% with F1 scores from 61.46% to 71.15%, P<0.05.

Ablation studies showed that classification performance declined when the post-BERT BiLSTM layer or the hybrid pooling of the BiLSTM outputs was omitted; moreover, to our surprise, when BERT-base was replaced by the BERT-large, the performance decreased. Error analysis suggested that longer documents, and RFPs with broad topics, were difficult for our model to correctly classify.

Although there are commercial systems for matching researchers to RFPs, [3], [4] our system is, to our knowledge, the first to provide personalized recommendations to a researcher (addressing limitation L2), to use deep learning (addressing limitation L1), and to rigorously and quantitatively evaluate an RFP-biosketch matching system (addressing limitation L3). Further, as mentioned in the introduction, existing commercial systems (e.g., Pivot or Elsevier Pure) seem to be based on (variations of) keyword matching, which is inflexible; our results reflect this as well, i.e., our baseline models that relied on bag of words and other features closely related to (key)word overlap performed much worse than the deep learning models. We therefore anticipate similar challenges that those existing commercial systems may face.

The relative advantages of each of our deep learning models, as well as the results of our ablation studies, are generally interpretable and/or consistent with previous work. For example, Siamese neural networks have been successfully applied in many text similarity measurement tasks, like Quora Question Pairs (QQP) [17], [37] but performed worse than the cross-encoders here. One explanation for this is that the RFP and the biosketch are not the same kind of document, so it may not be ideal to use the same model to process each of them independently, as in Siamese neural networks. Cross-encoding, however, avoids this problem, as it learns a representation not for each document separately, but for the combination of the documents. Likewise, we found that BiLSTM (on top of BERT) outperformed CNN. Previous work suggests that LSTM excels at extracting global features, while CNN excels at extracting local features [38], [39], [40]. Unlike our previous work, the stripped-down documents in this study, by keeping only “Personal Statement” of biosketches and the “Funding Opportunity Description” of RFPs, were still fairly lengthy (mean RFP length: 1,111 words; mean biosketch length: 393 words), and thus extracting global features is relatively more important (cf. classifying short documents like tweets [41], where extracting local information may be sufficient). Furthermore, given that the BERT model has a maximum input size of 512 tokens, we may need to devise a more effective strategy for addressing the challenge posed by a lengthy document. This could involve techniques like chunking or sliding windows to handle such cases more efficiently to achieve better document embeddings. Finally, our ablation studies are only partly consistent with other work. Previous work has shown the utility of having both transformer and other DNN components (BiLSTM or CNN) in a single architecture [42], [43], as well as the importance of using pooling strategies instead of directly utilizing final hidden states [44], [45]. More surprising is the superiority of BERT-large over BERT-base, given the superiority of the former in other NLP tasks [14]. For now, an explanation of this eludes us.

We were surprised that treating randomly sampled pairs as controls did not work effectively, when it worked very effectively for training models to match resumes with job descriptions. One possible reason is that job descriptions are more diverse in type of position (sales, software engineer, clerk) and industry (healthcare, entertainment, education, construction), whereas the RFPs focus entirely on one domain: health and life sciences. Thus, a randomly sampled RFP has a higher likelihood of being a match for a given biosketch, than does a randomly sampled job description of being a match for a resume, and hence it is less feasible to treat a random RFP as a control.

### A. LIMITATIONS

This study has limitations. Firstly, the dataset comprises only 420 manually annotated RFP-biosketch pairs, with biosketches from a single center (Children’s Hospital of Philadelphia) and RFPs from NIH. Secondly, the moderate inter-annotator agreement (Kappa scores around 50%) could be attributed to the complex and technical nature of the documents, covering diverse fields like medicine, engineering, and psychology. This variation potentially introduces noise into the labels, impacting model training (although we emphasize that this moderate agreement does not place a strict upper bound on model performance) [46]. Lastly, our focus was only on specific sections of the biosketches and RFPs, possibly omitting crucial information such as publications that could influence matchmaking accuracy.

### B. FUTURE DIRECTIONS

To address these limitations, future research will aim to develop a more extensive dataset incorporating RFPs and biosketches from a wider range of institutions and funding agencies, such as the Agency for Healthcare Research and Quality (AHRQ), the Department of Defense (DoD), and the National Science Foundation (NSF). Enhancing the annotation process is also a priority, potentially by matching annotators with documents based on their domain expertise to improve agreement and model performance. Further, expanding the scope of analysis to include complete documents may provide a more comprehensive view of the matchmaking process. Finally, evolving the model to offer a continuous match score and integrating explainable AI [47] could provide more nuanced and interpretable results, beneficial for both researchers and funders. The emerging role of Large Language Models (LLMs), such as LLaMA2, ChatGPT, etc., in natural language understanding presents an additional avenue for improving the accuracy of matchmaking between biosketches and specific RFPs.

## VI. CONCLUSION

Matching people to opportunities is a laborious task, suggesting automation may free up considerable resources. This problem is especially acute for researchers, who spend a substantial amount of time searching and applying for grants. To alleviate this bottleneck, here we developed and evaluated six machine-learning models to perform automated matching between RFPs and biosketches based on 420 annotated NIH RFP-biosketch pairs. Our BC2BT model using a cross-encoding architecture and BiLSTM layer trained with data augmentation had the best performance (F1-score 71.15%), outperformed baseline logistic regression and SVM models, as well as other deep neural network variants. We believe our work provides the groundwork for developing automated opportunity-worker matching systems – potentially with newly emerging LLM’s and in more diverse contexts than biomedical research, so that workers (e.g., researchers, reviewers, job seekers, etc.), and those providing them opportunities (e.g., funding agencies, journal editors, employers, etc.), can spend less time finding each other, and more time actually working with each other.

## Figures and Tables

**TABLE 1.** Four deep neural network models for matching between request for proposals and biosketches. These models were derived from two encoding strategies (cross-encoding and Siamese encoding) and two post-BERT layers (Bidirectional long short-term memory [BiLSTM] and Convolutional neural network [CNN]).

Post-BERT Layer	Encoding Strategy	
	Cross Encoding (CC)	Siamese Encoding (S)
Bi-LSTM	CC_BERT_BiLSTM	S_BERT_BiLSTM
CNN	CC_BERT_CNN	S_BERT_CNN

**TABLE 2.** Model performance of RFP-biosketch matching using four evaluation metrics in nested 10-fold cross validation: F1-score, Precision, Recall and Accuracy for each model with the 95% confidence interval (CI). Bold-faced numbers represent best performance. BERT_BiLSTM_Aug_de: used data augmentation with back translation (English→German→English); BERT_BiLSTM_Aug_ru: used data augmentation with back translation (English→Russian→English); *_Aug_de_ru: used both English↔German, and English↔Russian. All the BERT models in Table 1 used the base BERT except *CC_BERT_large_BiLSTM+Aug_de_ru* that used the large BERT.

Machine Learning Model	F1 % (95% C.I.)	Precision % (95% C.I.)	Recall % (95% C.I.)	Accuracy % (95% C.I.)
**Model without using Data Augmentation**				
Logistic Regression	51.14 (46.97–55.31)	51.65 (47.47–55.83)	51.51 (47.27–55.75)	52.43 (47.99–56.87)
SVM	55.13 (51.48–58.78)	55.99 (52.60–59.38)	56.13 (52.61–59.65)	55.80 (52.04–59.56)
S_BERT_CNN	50.21 (43.94–56.48)	48.63 (40.08–57.18)	54.28 (51.03–57.53)	59.64 (57.09–62.19)
S_BERT_BiLSTM	56.22 (53.01–59.43)	58.57 (53.81–63.33)	56.67 (53.37–59.97)	60.35 (56.84–63.86)
CC_BERT_CNN	60.92 (56.23–65.61)	63.61 (57.93–69.29)	60.96 (56.39–65.53)	64.45 (59.36–69.54)
CC_BERT_BiLSTM	61.46 (58.70–64.22)	62.98 (60.24–65.72)	61.64 (58.93–64.35)	65.33 (62.84–67.82)
**Models using Data Augmentation**				
CC_BERT_BiLSTM+Aug_de	68.95 (65.06–72.84)	70.48 (66.11–74.85)	68.58 (64.84–72.32)	71.22 (67.41–75.03)
CC_BERT_BiLSTM+Aug_ru	65.65 (56.70–74.60)	67.43 (56.27–78.59)	67.77 (61.98–73.56)	69.06 (61.73–76.39)
CC_BERT_large_BiLSTM+Aug_de_ru	69.36 (65.49–73.23)	72.03 (67.75–76.31)	68.99 (65.18–72.80)	72.20 (68.64–75.76)
CC_BERT_BiLSTM+Aug_de_ru (BC2BT)	**71.15 (66.34–75.96)**	**72.80 (67.51–78.09)**	**71.02 (66.18–75.86)**	**72.94 (68.13–77.75)**

**TABLE 3.** Ablation study of the effects of BERT embedding, feature extraction, and pooling.

	Actual match	Actual non-match
**Predicted Match**	101	54
**Predicted non-Match**	64	196

**TABLE 4.** Confusion matrix of the BC2BT model based on all 10 hold-out test sets.

Model	F1 score % (95% C.I.)	P-value
CC_BERT_BiLSTM+Aug_de_ru (BC2BT)	71.15 (66.34 – 75.96)	Ref.
**Ablation Test**		
Using only BERT embedding (large)	69.36 (65.49 – 73.23)	0.5770
Removing the feature extraction (BiLSTM) layer	64.47 (55.04 – 73.90)	0.2322
Removing the pooling layer	67.72 (64.44 – 71.00)	0.2632
